# A 10 year study of hospitalized atrial fibrillation-related stroke in England and its association with uptake of oral anticoagulation

**DOI:** 10.1093/eurheartj/ehy411

**Published:** 2018-07-05

**Authors:** J Campbell Cowan, Jianhua Wu, Marlous Hall, Andi Orlowski, Robert M West, Chris P Gale

**Affiliations:** 1 Department of Cardiology, Leeds Teaching Hospitals NHS Trust, Leeds General Infirmary, Great George Street, Leeds, UK; 2 Division of Clinical and Translational Research, School of Dentistry, University of Leeds, Leeds, UK; 3 Clinical and Population Science Department, Leeds Institute of Cardiovascular and Metabolic Medicine, University of Leeds, Leeds, UK; 4 Imperial College Health Partners, London, UK; 5 Leeds Institute of Health Sciences, University of Leeds, Leeds, UK; 6 Department of Cardiology, York Teaching Hospital NHS Foundation Trust, York, UK

**Keywords:** Atrial fibrillation, Anticoagulation, Stroke

## Abstract

**Aims:**

To determine whether changing patterns of anticoagulant use in atrial fibrillation (AF) have impacted on stroke rates in England.

**Methods and results:**

English national databases, 2006–2016, were interrogated to assess stroke admissions and oral anticoagulant use. The number of patients with known AF increased linearly from 692 054 to 983 254 (prevalence 1.29% vs. 1.71%). Hospital episodes of AF-related stroke/100 000 AF patients increased from 80/week in 2006 to 98/week in 2011 and declined to 86/week in 2016 (2006–2011 difference 18.0, 95% confidence interval (CI) 17.9–18.1, 2011–2016 difference −12.0, 95% CI −12.1 to −11.9). Anticoagulant use amongst patients with CHA_2_DS_2_-VASc ≥2 increased from 48.0% to 78.6% and anti-platelet use declined from 42.9% to 16.1%; the greatest rate of change occurred in the second 5 year period (for anticoagulants 2006–2011 difference 4.8%, 95% CI 4.5–5.1%, 2011–2016 difference 25.8%, 95% CI 25.5–26.1%). After adjustment for AF prevalence, a 1% increase in anticoagulant use was associated with a 0.8% decrease in the weekly rate of AF-related stroke (incidence rate ratio 0.992, 95% CI 0.989–0.994). Had the use of anticoagulants remained at 2009 levels, 4068 (95% CI 4046–4089) more strokes would have been predicted in 2015/2016.

**Conclusion:**

Between 2006 and 2016, AF prevalence and anticoagulant use in England increased. From 2011, hospitalized AF-related stroke rates declined and were significantly associated with increased anticoagulant uptake.

**Figure:**
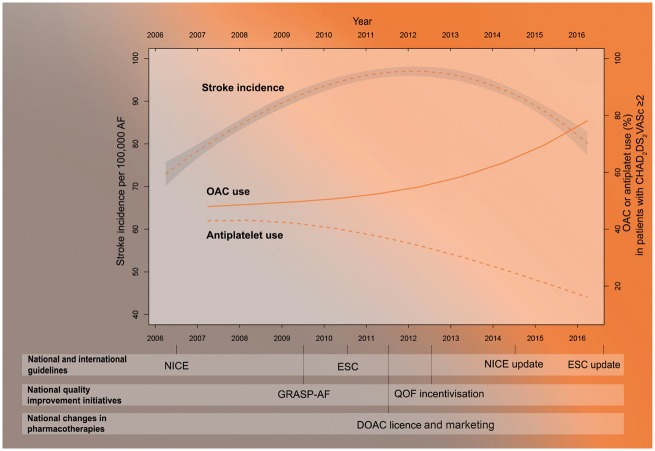

## Introduction

Stroke is a major cause of deaths due to cardiovascular disease. For strokes with an established aetiology, the substantial majority (87%) are known to be ischaemic.^1^ Approximately one-third of ischaemic strokes are associated with atrial fibrillation (AF).^2^ Strokes due to AF are of particular significance as they are preventable with anticoagulation.^3^ Recent international guidelines have lowered the threshold for the use of oral anticoagulants and encouraged their use in preference to anti-platelet drugs.^4–6^ Whether any resultant changes in prescribing practice have led to a reduction in AF-related stroke is unknown.

Existing evidence on temporal trends in AF-related stroke is conflicting. Data from earlier studies suggests a decline in strokes rates, relating to increasing oral anticoagulant uptake,^7^^,^^8^ but more recent studies have in general not indicated progressive benefit, perhaps due to a levelling off of oral anticoagulant uptake.^9–12^ A particular problem for all studies lies in defining the prevalence of known AF and oral anticoagulant uptake amongst the AF population at risk. This is especially important when both the prevalence and incidence of AF are known to be increasing.^13^^,^^14^

The United Kingdom is unique in that a range of national primary and secondary care databases exist, which have prospectively collected consistent data fields relating to clinical care and health outcomes over many years. We, therefore, used English multi-source electronic health records data to investigate changes in the prevalence of known AF in primary care and hospital admissions for AF-related stroke in England over 10 years between 2006 and 2016. Specifically, we aimed to ascertain whether any changes in oral anticoagulant uptake were associated with temporal changes in AF-related stroke.

## Methods

**Figure 1:**
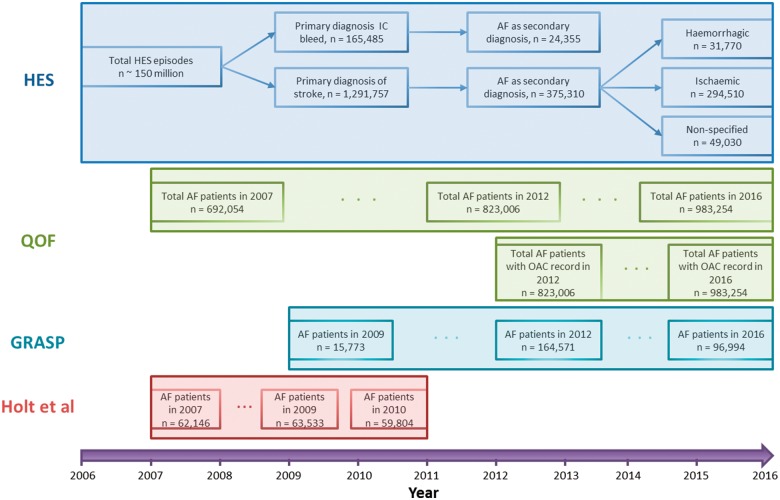
STROBE diagram of the derivation of the analytical cohorts and their associated timeframes. HES: Hospital Episode Statistics; QOF: Quality and Outcomes Framework; GRASP: Guidance on Risk Assessment for Stroke Prevention in AF; Holt *et al.*^20^

Temporal trends of AF-related stroke, standardized to AF prevalence, were compared with the uptake of oral anticoagulation over the 10 year study period from 2006 to 2016. No single national database containing all relevant information for this study exists. Therefore, a comprehensive set of aggregated national data was compiled (see below and *Figure 1*).

### Atrial fibrillation-related stroke

The number of episodes of hospitalized AF-related stroke in England were accessed from the Hospital Episode Statistics (HES) data warehouse.^15^ Aggregated weekly counts of the number of finished consultant episodes with a primary diagnosis of stroke (ischaemic, haemorrhagic, or unspecified) and secondary diagnosis of AF were extracted from inpatient HES data between 1 April 2006 and 31 March 2016. A finished consultant episode is the total time a patient spends under the care of an individual consultant. Since transfer to a different consultant team would count as a further episode, within each weekly period of record collection replicate episodes relating to an individual patient were eliminated.

The pathogenesis of stroke was defined according to the International Statistical Classification of Diseases and Related Health Problems (ICD-10) codes as ischaemic (I63.0-I63.9), haemorrhagic (I61.0-I61.9), or unspecified (I64.X). Strokes not coded as ischaemic or haemorrhagic were also categorized as unspecified. Atrial fibrillation was defined as ICD I48.X. The number of episodes of hospitalization with non-traumatic intracranial bleeding (ICD I60.0-I60.9, I62.0, I62.1 and I62.9) as a primary diagnosis and AF as a secondary diagnosis was also determined.

Charlson co-morbidity index categories I–III, based on myocardial infarction, heart failure, peripheral vascular disease, dementia, cerebrovascular disease, chronic lung disease, connective tissues disease, peptic ulcer disease, liver disease, diabetes, kidney disease, cancer, and HIV were calculated from HES data for all patients with a primary diagnosis of stroke and secondary diagnosis of AF.^16^^,^^17^

### Atrial fibrillation prevalence

The English national prevalence of AF was derived from the Clinical Domain of the Quality and Outcomes Framework (QOF) database for England (https://digital.nhs.uk/Quality-and-Outcomes-Framework/QOF). QOF is a national primary care database covering all primary care practices (*n* = 8372 practices for 2006/2007; *n* = 7619 for 2015/2016) in England and collects prevalence and treatment data for a number of conditions including AF. Data are gathered from individual practices at the end of March each year. De-identified QOF AF data, aggregated by practice and summarised at national level, were accessed from 1 April 2006 to 31 March 2016.

### Oral anticoagulation

De-identified patient data, aggregated to annual rates, concerning uptake of oral anticoagulants for patients living in England with a moderate to high risk of stroke measured as either a CHADS_2_ score ≥2 (1 April 2012 to 1 April 2015), or a CHA_2_DS_2_-VASc score ≥2 (1 April 2015 to 1 April 2016) were accessed from QOF.^18^^,^^19^ Given that prior to 1 April 2012 no single national data source was available for oral anticoagulant use, we used information from two large English cohorts to construct a timeline of national oral anticoagulant and anti-platelet drug use. This comprised (i) data from a published primary care series between 2007 and 2010 (99 351 patients from 583 general practices in England) and (ii) the Guidance on Risk Assessment for Stroke Prevention in AF (GRASP-AF) tool of the National Health Service Improving Quality (NHSIQ) and PRIMIS, University of Nottingham, between 2009 and 2016 (414 560 patients from 3162 general practices in England).^20^^,^^21^ These data were aggregated and weighted according to cohort size. In addition, information about the type of oral anticoagulant, vitamin K antagonist or direct oral anticoagulant (DOAC), was extracted for the GRASP-AF cohort between 2011 and 2016.

### Statistical analyses

Patient and clinical demographics for hospitalized AF-related stroke recorded in HES were described according to biennial period as well as overall between 2006 and 2016. Data from QOF, including the total number of AF cases and AF prevalence were described over the same time periods. Absolute differences alongside 95% confidence intervals (CIs) between the first and last biennial period were calculated for each variable.

The significance of linear time trends over the full period were assessed using logistic and linear regression models for categorical and continuous variables, respectively. In addition, we provide supplementary information on the differences in baseline variables between study periods calculated using the Difference-in-Difference (DiD) technique. Differential time effect and CIs were estimated using a fixed effect model for each baseline variable between two study periods (2006–2011 and 2011–2016).

The weekly rate of AF-related stroke per 100 000 patients with AF was standardized by dividing the weekly number of AF-related strokes by the weekly national AF prevalence derived from fitting a polynomial regression to the QOF annual data. The primary statistical analysis was based on weekly counts of hospitalized AF-related stroke. Although the weekly counts eliminate patient replicates within each week, they may reflect different stages in stroke management and not just the acute admission. To address this, we assessed stroke counts based on annual AF-related stroke admissions obtained from a second extract of HES data with the same specifications, but with aggregation to whole calendar years (2007–2015) and episode replicates eliminated over an annual timeframe.

To model the rate of hospitalized AF-related stroke and its association with the use of oral anticoagulants or anti-platelet drugs, a series of Poisson regression models were fitted. Initially, the unadjusted association of AF-related stroke with the percentage of oral anticoagulant usage was calculated in a univariate model. Subsequently, models were incrementally adjusted for temporal trends per week, including second and third degree polynomials of time to model non-linear associations. In addition, we included sine curves to model seasonality, as well as AF prevalence and patient demographics including sex, age in years, and Charlson Co-morbidity Index categories I to III. Results were presented as incidence rate ratios (IRRs) and 95% CIs.

To ascertain the potential influence of time variance in the reallocation of non-specified AF-related stroke pathology (because of, for example, changes in the use of cerebral imaging to enable greater diagnosis of ischaemic stroke), we conducted a sensitivity analysis of the above statistical analyses after re-classification of non-specified strokes to ischaemic and haemorrhagic strokes and compared the results with those of the main analyses.

All tests were two-sided, and statistical significance was considered *P* < 0.05. Statistical analyses were performed in R version 3.1.2 (https://cran.r-project.org/).

## Results

The AF-related stroke analytical cohort (*n* = 375 310) was drawn from 1 291 757 hospitalized finished consultant episodes with a primary diagnosis of stroke in England between 1 April 2006 and 31 March 2016 (*Figure 1*). These episodes were derived from the corresponding period of HES data for all hospitalizations in England (*n* = ∼150 million hospital episodes).^15^^,^^22^ Of all hospitalized finished consultant episodes of AF-related stroke, there were 294 510 (78.5%) ischaemic strokes, 31 770 (8.5%) haemorrhagic strokes, and 49 030 (13.0%) non-specified strokes. In total, there were 165 485 hospitalized episodes of intracranial bleeds of which 24 355 (14.9%) had a secondary diagnosis of AF. Of patients with AF-related stroke, 157 255 (41.9%) were men, mean age was 81.1 years, 100 891 (26.9%) were in the lowest (I), 32 879 (8.8%) in the middle (II), and 73 922 (19.7%) in the highest Charlson co-morbidity index category (*Table 1*). Temporal trends in co-morbidities of AF-related stroke are described in more detail in Supplementary material online, *Table S1*.

**Table 1:** Patient characteristics by period of study

Characteristic	Period of study							
	2006–2016	2006/2008^a^	2008/2010	2010/2012	2012/2014	2014/2016	Difference: 2014/2016– 2006/2008 (95% CI)	P-trend
Hospitalized finished consultant episodes of AF-related stroke^b^								
All stroke patients	375 310	57 874	69 817	78 795	84 159	84 665	26 791	0.014
Male	157 255 (41.9)	23 439 (40.5)	28 066 (40.2)	32 621 (41.4)	35 599 (42.3)	37 676 (44.5)	4.0 (3.5, 4.5)	0.025
Age (years), mean	81.12	80.74	81.12	81.12	81.17	81.35	0.61 (0.18, 1.04)	0.036
Deaths in hospital	57 360 (15.3)	10 741 (18.6)	11 713 (16.8)	11 606 (14.7)	11 703 (13.9)	11 597 (13.7)	−4.9 (-5.3,-4.5)	0.012
Charlson Comorbidity Index								
Category I	100 891 (26.9)	16 747 (28.9)	18 667 (26.7)	21 617 (27.4)	22 453 (26.7)	21 407 (25.3)	−3.7 (-4.1,-3.1)	0.055
Category II	32 879 (8.8)	4486 (7.8)	5441 (7.8)	6987 (8.9)	7931 (9.4)	8034 (9.5)	1.7 (1.9, 2.0)	0.015
Category III	73 922 (19.7)	9006 (15.6)	11 353 (16.3)	15 673 (19.9)	18 315 (21.8)	19 575 (23.1)	7.6 (7.1, 7.9)	0.003
Stroke classification								
Ischaemic	294 510	39 976	51 444	62 024	69 855	71 211	31 235	0.006
Haemorrhagic	31 770	4678	5504	6438	7179	7971	3293	<0.001
Intracranial bleeding	24 355	3381	4108	5086	5671	6109	2728	0.001
Quality and Outcomes Framework^c^								
Total AF	814 501	696 605	747 236	807 090	866 672	954 902	258 297	<0.001
Population AF prevalence (%)	1.43	1.29	1.37	1.46	1.54	1.67	0.38 (0.34, 0.42)	<0.001
Weekly stroke incidence per 100 000 AF	91	80	92	98	95	88	8 (7.9, 8.1)	0.47
Medication^d^								
Proportion of OAC use (%)	55.8	48.0	49.7	52.5	58.7	70.2	22.2 (22.1, 22.4)	<0.001
Proportion of antiplatelet drug use (%)	33.1	43.1	41.2	35.4	28.8	17.1	−26.0 (-26.6, 25.4)	<0.001

AF, atrial fibrillation; GRASP, Guidance on Risk Assessment and Stroke Prevention in AF; OAC, oral anticoagulants; QOF, Quality and Outcomes Framework.

a Two financial years between 1 April 2006 and 31 March 2008.

b For HES data, the accumulated number of AF-related stroke and percentage were reported unless specified.

c For QOF prevalence data, the mean estimated from the fitted regression model was reported for each period.

d Medication uptake data from Holt *et al.*,^20^ QOF, and GRASP was used. A weighted regression model was fitted to the use of OAC and antiplatelet drugs separately. The mean estimated from the regression model was reported for each period.

Over the 10 years, there were on average 814 501 cases of AF each year (prevalence 1.43%) across 7995 general practice surgeries in England. During this period, on average 57.2% patients with AF and a CHA_2_DS_2_-VASc score ≥2 received oral anticoagulation.

### Temporal trends in atrial fibrillation prevalence

**Figure 2:**
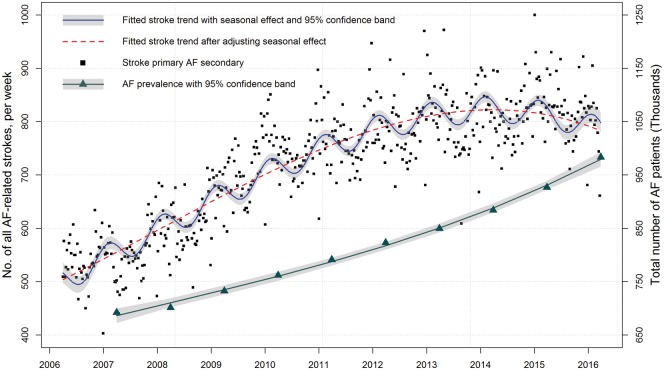
Temporal trend in finished consultant episodes of atrial fibrillation-related stroke and national prevalence of atrial fibrillation.

From 2006 to 2016, the number of patients with AF increased from 692 054 to 983 254 [prevalence 1.29% vs. 1.71%; absolute difference (AD) 0.42%, 95% CI 0.41–0.43%] (*Figure 2*, *Table 1*). The rate of increase in the prevalence of AF was similar in the first 5 year period 2006–2011 and the second 5 year period 2012–2016 (0.20%, 95% CI 0.16–0.24% vs. 0.22%, 95% CI 0.18–0.26%, Supplementary material online, *Table S2*).

### Temporal trends in atrial fibrillation-related stroke

**Figure 3:**
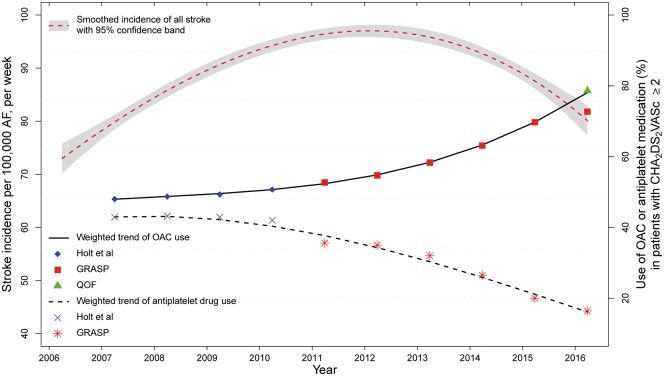
Temporal trend in finished consultant episodes of atrial fibrillation-related stroke per 100 000 patients with atrial fibrillation and uptake of oral anticoagulants and anti-platelet drugs for patients with atrial fibrillation and a CHA_2_DS_2_VASc score ≥2. The anticoagulant timeline is a weighted trend derived from Holt *et al.*,^20^ GRASP AF,^21^ and Quality and Outcomes Framework.

The numbers of hospitalized finished consultant episodes of AF-related stroke per week demonstrated regular changes each year in keeping with seasonality (*Figure 2*). Between 2006 and 2011, the rates of hospitalized finished consultant episodes of AF-related stroke per 100 000 patients with AF increased from 80 to 98 per week (difference 18.0, 95% CI 17.9–18.1) and declined to a rate of 86 per week in 2016 (difference 2011–2016: −12.0, 95% CI −12.1 to −11.9) (*Figure 3* and Supplementary material online, *Table S2*).

**Figure 4:**
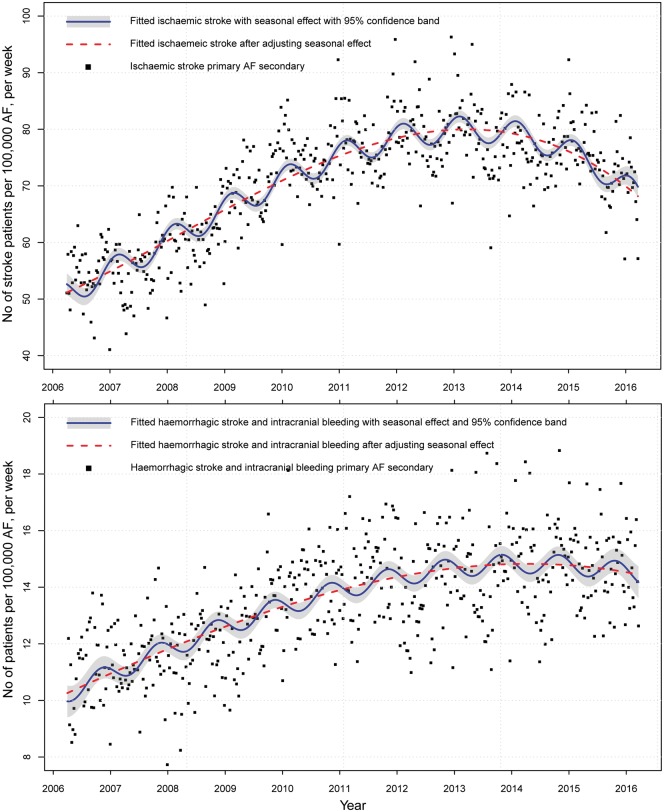
The temporal trend of atrial fibrillation-related stroke stratified by stroke pathogenesis.

The proportion of patients with AF-related stroke who were not classified as either ischaemic or haemorrhagic decreased from 24.5% in 2006 to 6.4% in 2016 (see Supplementary material online, *Figure S1*). The temporal trend in ischaemic stroke is presented in *Figure 4* together with the combined endpoint of haemorrhagic stroke and intracranial bleeding. Over the study period, there was a slight increase in the rates of the combined endpoint of cerebral haemorrhage and intracranial bleeding per 100 000 patients with AF (from 10.5 per week in 2006 to 14.4 per week in 2016, AD: 3.9 per week, 95% CI 3.6–4.2; 2011–2016 AD 0.4 per week, 95% CI 0.0–0.7, *Figure 4*).

### Temporal trends in the use of oral anticoagulants and antiplatelet drugs

**Figure 5:**
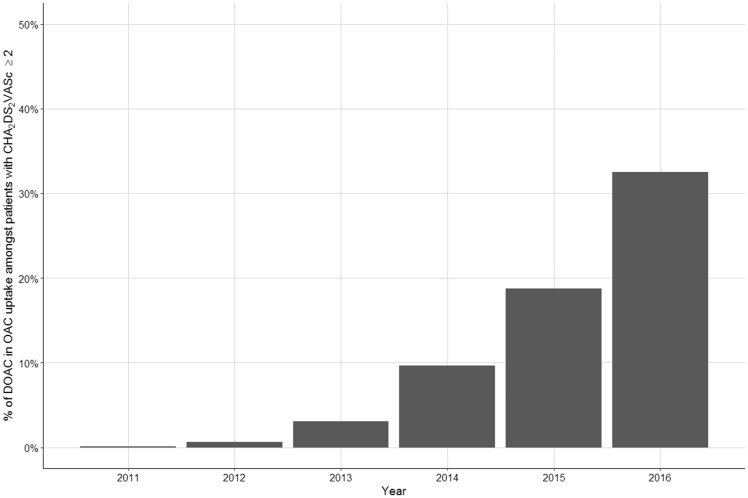
Direct oral anticoagulant uptake (as a percentage of all those taking an oral anticoagulant) amongst patients with CHA_2_DS_2_-VASc ≥2 in the GRASP cohort.

From 2006 to 2016, the use of oral anticoagulants among patients with AF and a CHA_2_DS_2_-VASc score ≥2 increased from 48.0% to 78.6% (AD: 30.6%, 95% CI 30.2–31.0%) (*Figure 3*) (For a comparison of CHADS_2_ and CHA_2_DS_2_-VASc based anticoagulant uptake, see Supplementary material online, *Figure S2*). The greatest rate of change in the use of anticoagulants occurred in the latter 5 year period (2006–2011 AD 4.8%, 95% CI 4.5–5.1%, 2011–2016 AD 25.8%, 95% CI 25.5–26.1%) (see Supplementary material online, *Table S2*). Amongst patients with CHA_2_DS_2_-VASc ≥2 in the GRASP cohort, the use of DOACs increased from 0.1% of the oral anticoagulant group in 2011 to 32.5% in 2016 (*Figure 5*).

Over the study period, the use of anti-platelet drugs among patients with AF and a CHA_2_DS_2_-VASc score ≥2 decreased from 42.9% to 16.1% (AD −26.8%, 95% CI −27.3% to −26.3%) (*Figure 3*). The greatest rate of change in the use of antiplatelet drugs for AF occurred in the latter 5 year period (2006–2011 AD −5.2%, 95% CI −5.5% to −4.9%, 2011–2016 AD −21.6%, 95% CI −22.1% to −21.1%) (see Supplementary material online, *Table S2*).

### Association between changing use of oral anticoagulants and anti-platelet drugs and atrial fibrillation-related stroke

Without adjustment, the use of oral anticoagulants demonstrated a significant 2.1% increase in finished consultant episodes of AF-related stroke (*Table 2*) (IRR 1.021, 95% CI 1.019–1.022). With adjustment for AF prevalence, sex, age, and co-morbidity, the effect was reversed suggesting that a 1% increase in the use of oral anticoagulants among patients with AF and a CHA_2_DS_2_-VASc score ≥2 was associated with a decline of 0.8% in weekly hospitalized AF-related strokes (IRR 0.992, 95% CI 0.989–0.994).

**Table 2:** Association between weekly rates of hospitalized finished consultant episodes of AF-related stroke and the use of oral anticoagulants, adjusted for AF prevalence and patient characteristics

Model	Use of oral anticoagulants (%)			AIC and BIC	
	Incident rate ratio (95% CI)	*P*-value	Absolute difference in rates (95% CI)	AIC	BIC
Unadjusted use of OAC	1.021 (1.019–1.022)	<0.001	0.021 (0.019 to 0.021)	11 749.2	11 757.7
Use of OAC adjusted for					
AF^	0.955 (0.954–0.958)	<0.001	−0.045 (−0.046 to −0.043)	6222.6	6235.3
AF, sex, and age	0.959 (0.957–0.961)	<0.001	−0.041 (−0.045 to −0.041)	6142.6	6163.8
AF, sex, age, and CCI	0.992 (0.989–0.994)	<0.001	−0.008 (−0.011 to −0.006)	5120.5	5154.5

AF, atrial fibrillation; AF^ refers to total national AF prevalence derived from QOF data; AIC, Akaike Information Criteria; BIC, Bayesian Information Criteria; CCI, Charlson Comorbidity Index; OAC, oral anticoagulants.

When the use of anti-platelet drugs was modelled rather than the use of oral anticoagulants (see Supplementary material online, *Table S3*), there was a negative association between the use of anti-platelet drugs and stroke (IRR 0.980, 95% CI 0.979–0.981). With adjustment for AF prevalence, sex, age, and co-morbidity, this effect was reversed (IRR 1.033, 95% CI 1.028–1.038).

### Sensitivity analyses

To determine the impact of the temporal changes in the decrease of unspecified diagnoses, we conducted a sensitivity analyses in which 10% of the unspecified diagnoses were reallocated as ‘haemorrhagic’ and 90% were reallocated as ‘ischaemic’. This analysis did not alter the direction or magnitude of the final model estimates (IRR 0.996, 95% CI 0.994–0.998) (see Supplementary material online, *Tables S4* and *S5*, *Figure S3*).

When AF-related stroke rates were considered over an annual time frame (see Supplementary material online, *Table S6 andFigures 4*) and the ratio of the annual sum of weekly strokes to the annual timeframe was calculated, the ratio did not show a significant trend with time.

### Magnitude of anticoagulant benefit

The number of hospitalized AF-related strokes in 2015/16 was 42 296 (95% CI 41 663–42 929). This was estimated to represent 4, 068 (95% CI 4046–4089) fewer hospitalized AF-related strokes than would have been predicted had oral anticoagulation rates remained at the 2009 level (49% in 2009 vs. 79% in 2015/16) (For details of the calculation, see Supplementary material online, *Section 2*).

## Discussion

In this analysis of national multi-source electronic health records between 2006 and 2016, we found that an increase in the nationwide uptake of oral anticoagulants in patients with AF and a CHA_2_DS_2_-VASc score ≥2 was significantly associated with a decline in hospitalized AF-related stroke.

To date, time course studies of temporal changes in AF-related stroke have reached differing conclusions. Data from the Framingham population found a decline in risk of stroke occurring following the onset of AF between 1958 and 2007.^7^ Data from Medicare between 1992 and 2007 also reported a decline in stroke rates amongst patients with AF, coinciding with a doubling of oral anticoagulant uptake.^8^ In contrast, another US study found that rates of stroke and transient ischaemic attacks remained unchanged between 2000 and 2010, which was attributed to a plateauing of oral anticoagulant use.^9^ A UK study found no reduction in AF-related stroke and other embolic vascular events between 2002 and 2012.^10^ Two Asian studies have shown either a progressive rise in AF-related stroke or a biphasic trend.^11^^,^^12^ Evidence from the SENTINEL database of stroke admissions in England, between 2013 and 2017, showed the proportion of strokes with known antecedent AF was constant at approximately 20%, despite a rising proportion receiving prior oral anticoagulation.^23^

Our study extends previous investigations by combining national information on incident stroke with the national prevalence of known AF. From 2011 to 2016, we found a nationwide shift in clinical practice reflected in an increased use of oral anticoagulants and a corresponding decrease in the use of anti-platelet drugs among patients with AF and a CHA_2_DS_2_-VASc score ≥2. While it is not possible to attribute this change in practice to a single cause, guideline changes, quality improvement initiatives and the advent of DOACs may have been contributory. Notably, European Society of Cardiology (ESC) and NICE guidelines were published and subsequently revised during the course of this study, with decreasing emphasis or complete removal of recommendations for the use of antiplatelet drugs amongst lower risk patients.^4–6^ In the UK, evidence for the underutilisation of oral anticoagulants led to national quality improvement initiatives to improve their uptake.^20^^,^^21^^,^^24^ For example, GRASP-AF was implemented as a national service improvement tool in 2009 to improve oral anticoagulant uptake.^21^ In 2012, changes to QOF incentivised general practitioners to use oral anticoagulants in preference to anti-platelet agents for patients with a CHADS_2_ score ≥2. In addition the advent of DOACs, together with the publicity and marketing associated with their introduction, may have contributed to increased uptake of oral anticoagulants. The first DOAC agent was licensed for stroke prophylaxis in AF in the UK in 2011. Following their introduction, the use of DOACs increased progressively, coinciding with the increase in oral anticoagulant uptake in the second half of the study period. A similar progressive rise in DOAC uptake has been reported by other studies.^25^^,^^26^ Increasing DOAC use may also contribute to increased oral anticoagulant uptake through increased therapy persistence in comparison with the use of vitamin K antagonists.^25^

**Take home figure:**
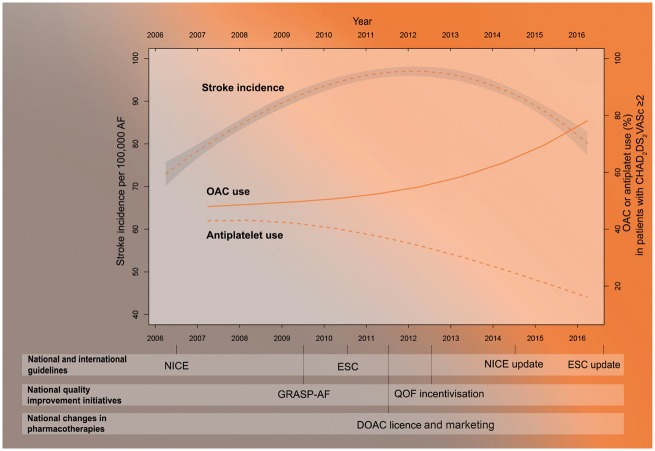


Direct oral anticoagulants reduce the frequency of cerebral haemorrhage and intracranial bleeding in comparison with warfarin.^27^ Although a small increase in intracranial bleeding and haemorrhagic stroke was observed in the 10 year period overall, there was no significant change in the latter 5 year period when the increase in uptake of oral anticoagulants was most pronounced. This may reflect the increasing use of DOACs.

Whilst this study has strengths such as single national health system coverage over a decade, we acknowledge its limitations. The data were aggregated and not linked, and we were unable to track individual patients or include all possible confounding factors over the study period. This aggregation could lead to a potential ‘ecological fallacy’ in which the findings are attributed incorrectly to the individual patient level; that is, one cannot assume that our findings are applicable to individual patients. In addition, we only had co-morbidity data for cases of AF-related stroke and not for the population at risk. Our primary outcome of hospitalized AF-related stroke will not have counted cases of stroke that were managed in the community or cases that died prior to hospitalization. We had no information on the timing of the AF identified as a secondary diagnosis. Our analysis was based on weekly rates of finished consultant episodes rather than unique hospital admissions with stroke, although a sensitivity analysis over an annual timeframe suggested that the observed temporal trends reflected stroke incidence. Although more stroke patients received diagnostic imaging in the latter years, a sensitivity analysis exploring this did not change the overall study conclusions.

The uptake of oral anticoagulants for stroke prophylaxis varies internationally and tends, for example, to be higher in Europe and Japan than in the USA and the rest of Asia.^3^^,^^28^ Our analysis suggests that for communities with modest anticoagulant uptake, increasing uptake offers considerable potential for stroke reduction.^29^ For communities already achieving a high level of oral anticoagulant use, the potential for further gains through increasing uptake can only wane due a ‘ceiling effect’ from a residual core of patients with contraindications. In these communities, particular attention to enhancing identification of unrecognized AF, whether through improving clinical detection or electrocardiogram monitoring strategies,^30^ may be justified.

In conclusion, we have observed an association between an increase in oral anticoagulant uptake and a decline in AF prevalence corrected stroke rate in England. While our study reports only a descriptive association and cannot demonstrate causality, our analysis suggests that increase in anticoagulant uptake contributed to a reduction of some 4000 strokes in England in 2015/2016 in comparison with projected stroke rates if the use of oral anticoagulants had remained constant at 2009 levels.

## Ethical approval

We used de-identified unlinked data aggregated to national level to preclude the reporting of potentially identifiable small counts. Therefore, according to Section 2.3 of the 2011 Governance Arrangements for Research Ethics Committees (GAfREC) this study did not require a favourable ethical opinion.

## Supplementary Material

Supplementary Data
