# Supplementary material for: A 10 year study of hospitalized atrial fibrillation-related stroke in England and its association with uptake of oral anticoagulation
Source: Eur Heart J. 2018 Jul 5;39(32):2975–83. doi: 10.1093/eurheartj/ehy411 (PMC6110195; doi:10.1093/eurheartj/ehy411)
Supplement: Supplementary Data [file ehy411_supplementary_materials.docx]

**Supplementary materials**

**Title: A 10 year study of hospitalised atrial fibrillation-related stroke in England and its association with uptake of oral anticoagulation**

**Supplementary section 1:**

***Temporal trends in co-morbidities of AF-related stroke***

Between 2006 and 2016, there was an increase in the proportion of cases of hospitalised AF-related stroke with higher levels of co-morbidity (Charlson Co-morbidity Index category III 15·6% vs. 23·9%) and decline in those with lower levels of co-morbidity (Charlson Co-morbidity Index category I 29·4% vs. 25·0%) However, the rate of change was less in the latter 5 year period compared with the former 5 year period (Charlson Co-morbidity Index category III: 2006-2011 difference 4·7%, 95% CI 4·4 to 5·2%, 2011-2016 difference 3·5%, 95% CI 3·0 to 4·0%; DiD -1·2%, 95% CI -2·2 to -0·3%; Charlson Co-morbidity Index category I: 2006-2011 difference -1·5%, 95% CI -2·0 to -1·0%, 2011-2016 difference -2·9%, 95% CI -3·4 to -2·4%; DiD -1·4%, 95% CI -2·5 to -0·3%) (Supplementary Table 1). The co-morbidity with the greatest increase in frequency between 2006 and 2016 was renal disease (2·6% vs. 7·4%, p<·001; AD 4·8%, 95% CI 4·6 to 5·0%) followed by diabetes mellitus (7·9% vs. 11·7%, p =·002; AD 3·8%, 95% CI 3·5 to 4·1%) whereas none of the remaining Charlson Co-morbidity Index disease categories declined in frequency (Supplementary Table 1).

**Supplementary table 1. Patient characteristics including comorbidities by period of study.**

|  | **Period of study** | | | | | | | |
| --- | --- | --- | --- | --- | --- | --- | --- | --- |
| **Characteristic** | **2006-2016** | **2006/2008^a^** | **2008/2010** | **2010/2012** | **2012/2014** | **2014/2016** | **Difference:**  **2014/2016-2006/2008**  **(95% CI)** | **P-trend** |
| **Hospitalised finished consultant episodes of AF-related stroke^b^** | | | |  |  |  |  |  |
| All stroke patients | 375310 | 57874 | 69817 | 78795 | 84159 | 84665 | 26791 | 0.014 |
| Male | 157255 (41.9) | 23439 (40.5) | 28066 (40.2) | 32621 (41.4) | 35599 (42.3) | 37676 (44.5) | 4.0 (3.5,4.5) | 0.025 |
| Age in years, mean | 81.12 | 80.74 | 81.12 | 81.12 | 81.17 | 81.35 | 0.61 (0.18,1.04) | 0.036 |
| Deaths in hospital | 57360 (15.3) | 10741 (18.6) | 11713 (16.8) | 11606 (14.7) | 11703 (13.9) | 11597 (13.7) | -4.9 (-5.3,-4.5) | 0.012 |
| Charlson Comorbidity Index |  |  |  |  |  |  |  |  |
| Category I | 100891 (26.9) | 16747 (28.9) | 18667 (26.7) | 21617 (27.4) | 22453 (26.7) | 21407 (25.3) | -3.7 (-4.1,-3.1) | 0.055 |
| Category II | 32879 (8.8) | 4486 (7.8) | 5441 (7.8) | 6987 (8.9) | 7931 (9.4) | 8034 (9.5) | 1.7 (1.9,2.0) | 0.015 |
| Category III | 73922 (19.7) | 9006 (15.6) | 11353 (16.3) | 15673 (19.9) | 18315 (21.8) | 19575 (23.1) | 7.6 (7.1,7.9) | 0.003 |
| Acute myocardial infarction | 23669 (6.3) | 2923 (5.1) | 3909 (5.6) | 5520 (7.0) | 5848 (6.9) | 5469 (6.5) | 1.4 (1.1,1.7) | 0.123 |
| Congestive heart failure | 25076 (6.7) | 3079 (5.3) | 3812 (5.5) | 5166 (6.6) | 6260 (7.4) | 6759 (8.0) | 2.7 (2.4,3.0) | 0.002 |
| Connective tissue disorder | 5425 (1.4) | 676 (1.2) | 793 (1.1) | 1164 (1.5) | 1344 (1.6) | 1447 (1.7) | 0.5 (0.4,0.6) | 0.029 |
| Dementia | 18426 (4.9) | 1878 (3.2) | 2579 (3.7) | 3666 (4.7) | 4736 (5.6) | 5567 (6.6) | 3.3 (3.2,3.6) | <0.001 |
| Diabetes | 37446 (10.0) | 4603 (8.0) | 5838 (8.4) | 7800 (9.9) | 9283 (11.0) | 9922 (11.7) | 3.8 (3.4,4.0) | 0.002 |
| Liver disease | 1252 (0.3) | 165 (0.3) | 140 (0.2) | 266 (0.3) | 356 (0.4) | 326 (0.4) | 0.1 (0.03,0.2) | 0.139 |
| Peptic ulcer | 2262 (0.6) | 406 (0.7) | 421 (0.6) | 471 (0.6) | 482 (0.6) | 483 (0.6) | -0.1 (-0.2,-0.01) | 0.182 |
| Peripheral vascular disease | 8394 (2.2) | 1055 (1.8) | 1240 (1.8) | 1676 (2.1) | 2156 (2.6) | 2266 (2.7) | 0.9 (0.7,1.1) | 0.011 |
| Pulmonary disease | 23053 (6.1) | 2680 (4.6) | 3326 (4.8) | 4813 (6.1) | 5669 (6.7) | 6565 (7.8) | 3.1 (2.9,3.5) | 0.003 |
| Cancer | 8081 (2.2) | 927 (1.6) | 1274 (1.8) | 1653 (2.1) | 2003 (2.4) | 2223 (2.6) | 1.0 (0.8,1.2) | <0.001 |
| Diabetes complications | 2548 (0.7) | 416 (0.7) | 466 (0.7) | 541 (0.7) | 547 (0.6) | 578 (0.7) | -0.04 (-0.09,0.09) | 0.559 |
| Hemiplegia (paraplegia) | 12240 (3.3) | 1956 (3.4) | 1834 (2.6) | 1861 (2.4) | 1517 (1.8) | 5071 (6.0) | 2.6 (2.4,2.8) | 0.478 |
| Renal disease | 19004 (5.1) | 1626 (2.8) | 2608 (3.7) | 3796 (4.8) | 4994 (5.9) | 5980 (7.1) | 4.3 (4.1,4.5) | <0.001 |
| Metastatic cancer | 2817 (0.8) | 481 (0.8) | 499 (0.7) | 557 (0.7) | 616 (0.7) | 664 (0.8) | -0.05 (-0.1,0.1) | 1 |
| Severe liver disease | 541 (0.1) | 80 (0.1) | 60 (0.1) | 145 (0.2) | 145 (0.2) | 110 (0.1) | -0.01 (-0.04,0.04) | 0.638 |
| HIV | 55 (0.0) | 0 (0) | 5 (0.0) | 5 (0.0) | 25 (0.0) | 20 (0.0) | - | - |
| Abbreviations: AF, atrial fibrillation; GRASP, Guidance on Risk Assessment and Stroke Prevention.  a Two financial years between 01/04/2006 and 31/03/2008; | | | | | |  |  |  |
| b For HES data, the accumulated number of AF-related stroke and percentage were reported unless specified; | | | | | | |  |  |

**Supplementary Table 2. Characteristics of patients from HES, QOF and GRASP and 5-year differences.**

|  | **Period of study** | | | | | |
| --- | --- | --- | --- | --- | --- | --- |
| **Characteristic** | **2006/2007^a^** | **2010/2011** | **2015/2016** | **Difference:**  **2006-2011 (95% CI)** | **Difference:**  **2011-2016 (95% C)** | **Difference in difference**  **(95% CI)** |
| **Hospitalised finished consultant episodes of AF-related stroke^b^** | | | |  |  |  |
| Total patients | 28203 | 40011 | 42060 | 11808 | 2049 | -9759 |
| Male (%) | 11338 (40·2) | 16725 (41·8) | 19011 (45·2) | 1·6 (1·1,2·1) | 3·4 (2·6,3·9) | 1·8 (0·6,3·0) |
| Age in years, mean | 80·65 | 81·14 | 81·31 | 0·49 (0·06,0·93) | 0·17 (-0·26,0·60) | -0·32 (-1·29,0·65) |
| Deaths in hospital | 5408 (19·2) | 5763 (14·4) | 5677 (13·5) | -4·8 (-5·2,-4·4) | -0·9 (-1·3,-0·5) | 3·9 (3·0,4·8) |
| Charlson Comorbidity Index |  |  |  |  |  |  |
| Category I | 8305 (29·4) | 11182 (27·9) | 10518 (25·0) | -1·5 (-2·0,-1·0) | -2·9 (-3·4,-2·4) | -1·4 (-2·5,-0·3) |
| Category II | 2204 (7·8) | 3710 (9·3) | 3829 (9·1) | 1·5 (1·2,1·8) | -0·2 (-0·5,0·1) | -1·6 (-2·3,-0·9) |
| Category III | 4410 (15·6) | 8155 (20·4) | 10046 (23·9) | 4·7 (4·4,5·2) | 3·5 (3·0,4·0) | -1·2 (-2·2,-0·3) |
| Acute myocardial infarction | 1444 (5·1) | 2857 (7·1) | 2727 (6·5) | 2·0 (1·7,2·3) | -0·7 (-0·9,-0·3) | -2·7 (-3·3,-2·1) |
| Congestive heart failure | 1489 (5·3) | 2716 (6·8) | 3515 (8·4) | 1·5 (1·2,1·8) | 1·6 (1·3,1·9) | 0·06 (-0·6,0·7) |
| Connective tissue disorder | 330 (1·2) | 605 (1·5) | 719 (1·7) | 0·3 (0·2,0·4) | 0·2 (0·06,0·3) | -0·1 (-0·4,0·2) |
| Dementia | 928 (3·3) | 1943 (4·9) | 2842 (6·8) | 1·6 (1·4,1·8) | 1·9 (1·6,2·2) | 0·3 (-0·2,0·9) |
| Diabetes | 2229 (7·9) | 4112 (10·3) | 4921 (11·7) | 2·4 (2·1,2·7) | 1·4 (1·1,1·7) | -1·0 (-1·7,-0·2) |
| Liver disease | 70 (0·2) | 120 (0·3) | 160 (0·4) | 0·05 (0·04,0·2) | 0·1 (0·03,0·2) | 0·03 (-0·1,0·2) |
| Peptic ulcer | 195 (0·7) | 225 (0·6) | 257 (0·6) | -0·1 (-0·2,-0·01) | 0·05 (-0·1,0·1) | 0·2 (-0·01,0·4) |
| Peripheral vascular disease | 511 (1·8) | 878 (2·2) | 1165 (2·8) | 0·4 (0·2,0·6) | 0·6 (0·4,0·8) | 0·2 (-0·2,0·6) |
| Pulmonary disease | 1288 (4·6) | 2590 (6·5) | 3319 (7·9) | 1·9 (1·6,2·2) | 1·4 (1·1,1·7) | -0·5 (-1·1,0·1) |
| Cancer | 444 (1·6) | 880 (2·2) | 1130 (2·7) | 0·6 (0·4,0·8) | 0·5 (0·3,0·7) | -0·1 (-0·5,0·2) |
| Diabetes complications | 215 (0·8) | 276 (0·7) | 292 (0·7) | -0·08 (-0·2,-0·01) | 0·01 (-0·09,0·09) | 0·08 (-0·1,0·3) |
| Hemiplegia (Paraplegia) | 997 (3·5) | 820 (2·0) | 4231 (10·1) | -1·5 (-1·7,-1·3) | 8·0 (7·8,8·4) | 9·5 (9·0,9·9) |
| Renal disease | 739 (2·6) | 1988 (5·0) | 3108 (7·4) | 2·3 (2·2,2·6) | 2·4 (2·1,2·7) | 0·07 (-0·5,0·6) |
| Metastatic cancer | 245 (0·9) | 281 (0·7) | 352 (0·8) | -0·2 (-0·3,-0·1) | 0·1 (0·0,0·2) | 0·3 (0·09,0·5) |
| Severe liver disease | 45 (0·2) | 45 (0·1) | 70 (0·2) | -0·05 (-0·1,-0·04) | 0·05 (0·04,0·15) | 0·1 (0·01,0·2) |
| HIV | 0 (0) | 5 (0·0) | 15 (0·0) | - | - | - |
| **Stroke classification** |  |  |  |  |  |  |
| Ischaemic | 19039 | 32067 | 35354 | 13028 | 3287 | -9741 |
| Haemorrhagic | 2286 | 3294 | 4086 | 1008 | 792 | -216 |
| Intracranial bleeding | 1585 | 2599 | 3077 | 1014 | 478 | -536 |
| **Quality and Outcomes Framework^c^** |  |  |  |  |  |  |
| Total AF | 692054 | 823006 | 983254 | 127136 | 148049 | 20913 |
| Population AF prevalence (%) | 1·29 | 1·49 | 1·71 | 0·20 (0·16,0·24) | 0·22 (0·18,0·26) | 0·02 (-0·04,0·08) |
| Weekly stroke incidence per 100,000 AF | 80 | 98 | 86 | 18 (17·9,18·1) | -12 (-12·1,-11·9) | -30 (-30·1,-29·9) |
| **Medication** |  |  |  |  |  |  |
| Proportion of OAC use (%) | 48·0 | 52·8 | 78·6 | 4·8 (4·5,5·1) | 25·8 (25·5,26·1) | 21·0 (20·6,21·4) |
| Proportion of antiplatelet drug use (%) | 42·9 | 37·7 | 16·1 | -5·2 (-5·5, -4·9) | -21·6 (-22·1,-21·1) | -16·4 (-16·8,-15·9) |
| Abbreviations: AF, atrial fibrillation; OAC, oral anticoagulants; QOF, Quality Outcomes Framework; GRASP, Guidance on Risk Assessment and Stroke Prevention.  a Financial year between 01/04/2006 and 31/03/2007; | | | | | |  |
| b For HES data, the accumulated number of AF-related stroke and percentage were reported unless specified;  c For QOF data, the value at the end of the financial year was reported except for weekly stroke incidence per 100,000 AF, where the average weekly incidence was reported.  d Medication uptake data for patients with AF and a CHA_2_DS_2_VASc score ≥ 2 from Holt et al., QOF and GRASP was used. A weighted regression model was fitted to the use of OAC and antiplatelet drug separately. The point estimate of uptake at the end of the financial year was reported for each period.  Differences in baseline variables between study periods were calculated using the Difference-in-Difference (DiD) technique. | | | | | | |

**Supplementary Table 3.**

**Association between weekly rates of hospitalised finished consultant episodes of AF-related stroke and the use of antiplatelet drugs for patients with AF and a CHA_2_DS_2_-VASc score ≥ 2, adjusted for AF prevalence and patient characteristics.**

|  | Use of antiplatelet drugs | | |  | AIC and BIC | |
| --- | --- | --- | --- | --- | --- | --- |
| Model | Incident rate ratio  (95% CI) | P Value | Absolute difference in rates  (95% CI) |  | AIC | BIC |
| Unadjusted use of antiplatelet drugs | 0·980 (0·979,0·981) | <·001 | -0·021 (-0·021,-0·020) |  | 9810·4 | 9818·9 |
| Use of antiplatelet drugs adjusted for |  |  |  |  |  |  |
| AF^ | 1·095 (1·091,1·100) | <·001 | 0·091 (0·087,0·095) |  | 6542·1 | 6554·8 |
| AF, sex, and age | 1·091 (1·086,1·095) | <·001 | 0·087 (0·083,0·091) |  | 6443·7 | 6465·0 |
| AF, sex, age, and CCI | 1·033 (1·028,1·038) | <·001 | 0·032 (0·027,0·037) |  | 4983·3 | 5017·3 |

Abbreviation: OAC, oral anticoagulants; AF, atrial fibrillation; CCI, Charlson Comorbidity Index;
AIC, Akaike Information Criteria; BIC, Bayesian Information Criteria.
AF^ refers to total national AF prevalence derived from QOF data

**Supplementary Table 4.**

**Sensitivity analysis for the association between weekly rates of hospitalised finished consultant episodes of AF-related ischaemic stroke combined with 90% unspecified stroke and the use of oral anticoagulants for patients with AF and a CHA_2_DS_2_-VASc score ≥2, adjusted for AF prevalence and patient characteristics.**

|  | Use of OAC | | |  | AIC and BIC | |
| --- | --- | --- | --- | --- | --- | --- |
| Model | Incident rate ratio  (95% CI) | P Value | Absolute Difference in rates  (95% CI) |  | AIC | BIC |
| Unadjusted use of OAC | 1·013 (1·012,1·014) | <·001 | 0·013 (0·012,0·014) |  | 10057·4 | 10065·9 |
| Use of OAC adjusted for |  |  |  |  |  |  |
| AF^ | 0·966 (0·964,0·967) | <·001 | -0·035 (-0·037,-0·033) |  | 6564·3 | 6577·1 |
| AF, sex, and age | 0·967 (0·966,0·969) | <·001 | -0·033 (-0·035,-0·032) |  | 6478·3 | 6499·6 |
| AF, sex, age, and CCI | 0·996 (0·994,0·998) | <·001 | -0·004 (-0·007,-0·002) |  | 5202·7 | 5236·7 |

Abbreviation: OAC, oral anticoagulants; AF, atrial fibrillation; CCI, Charlson comorbidity index;
AIC, Akaike Information Criteria; BIC, Bayesian Information Criteria.
AF^ refers to total national AF prevalence derived from QOF data

**Supplementary Table 5.**

**Sensitivity analysis for the association between weekly rates of hospitalised finished consultant episodes of AF-related ischaemic stroke combined with 90% unspecified stroke and the use of antiplatelet drugs for patients with AF and a CHA_2_DS_2_-VASc score ≥2, adjusted for AF prevalence and patient characteristics.**

|  | Use of antiplatelet drugs | | |  | AIC and BIC | |
| --- | --- | --- | --- | --- | --- | --- |
| Model | Incident rate ratio  (95% CI) | P Value | Absolute Difference in rates  (95% CI) |  | AIC | BIC |
| Unadjusted use of antiplatelet drugs | 0·987 (0·986,0·988) | <·001 | -0·013 (-0·014,-0·012) |  | 8999·9 | 9008·4 |
| Use of antiplatelet drugs adjusted for |  |  |  |  |  |  |
| AF^ | 1·074 (1·071,1·078) | <·001 | 0·072 (0·068,0·075) |  | 6674·0 | 6686·7 |
| AF, sex, and age | 1·070 (1·066,1·074) | <·001 | 0·068 (0·064,0·072) |  | 6582·6 | 6603·9 |
| AF, sex, age, and CCI | 1·024 (1·020,1·028) | <·001 | 0·024 (0·020,0·028) |  | 5090·4 | 5124·4 |

Abbreviation: OAC, oral anticoagulants; AF, atrial fibrillation; CCI, Charlson Comorbidity Index;
AIC, Akaike Information Criteria; BIC, Bayesian Information Criteria.
AF^ refers to total national AF prevalence derived from QOF data

**Supplementary Table 6.**

**Annual sum of weekly sampling timeframe counts and annual timeframe sampling counts of AF related stroke, presented by study year.**

| **Classification** | **Study period** | | | | | | | | | |  |
| --- | --- | --- | --- | --- | --- | --- | --- | --- | --- | --- | --- |
|  | **2007** | **2008** | **2009** | **2010** | **2011** | **2012** | **2013** | **2014** | **2015** | **P-Trend** | |
| All AF-related stroke |  |  |  |  |  |  |  |  |  |  | |
| Weekly count | 28520 | 31608 | 36305 | 38819 | 39964 | 41095 | 41915 | 42727 | 42475 | <0.001 | |
| Annual count | 18129 | 20168 | 22538 | 23796 | 24864 | 25873 | 26196 | 26387 | 26235 | 0.001 | |
| Ratio of weekly to annual count | 1.573 | 1.567 | 1.611 | 1.631 | 1.607 | 1.588 | 1.600 | 1.619 | 1.619 | 0.104 | |
| AF-related ischaemic stroke |  |  |  |  |  |  |  |  |  |  | |
| Weekly count | 19898 | 22758 | 26952 | 29603 | 31671 | 33741 | 34967 | 35909 | 35759 | <0.001 | |
| Annual count | 12626 | 14542 | 16735 | 18465 | 19897 | 21315 | 21840 | 22143 | 22022 | <0.001 | |
| Ratio of weekly to annual count | 1.576 | 1.565 | 1.611 | 1.603 | 1.592 | 1.583 | 1.601 | 1.622 | 1.624 | 0.126 | |
| AF-related haemorrhagic stroke |  |  |  |  |  |  |  |  |  |  | |
| Weekly count | 2298 | 2484 | 2865 | 3163 | 3307 | 3362 | 3584 | 3890 | 3923 | <0.001 | |
| Annual count | 1538 | 1670 | 1931 | 2024 | 2145 | 2255 | 2357 | 2512 | 2705 | <0.001 | |
| Ratio of weekly to annual count | 1.494 | 1.487 | 1.484 | 1.563 | 1.542 | 1.491 | 1.521 | 1.549 | 1.450 | 0.970 | |
| AF-related unspecified stroke |  |  |  |  |  |  |  |  |  |  | |
| Weekly count | 6379 | 6435 | 6570 | 6135 | 5078 | 4085 | 3446 | 3031 | 2859 | <0.001 | |
| Annual count | 4458 | 4526 | 4628 | 4076 | 3497 | 2925 | 2519 | 2270 | 2041 | <0.001 | |
| Ratio of weekly to annual count | 1.431 | 1.422 | 1.420 | 1.505 | 1.452 | 1.397 | 1.368 | 1.335 | 1.401 | 0.120 | |
| AF-related intracranial bleeding |  |  |  |  |  |  |  |  |  |  | |
| Weekly count | 1803 | 1900 | 2063 | 2390 | 2663 | 2619 | 2834 | 3076 | 3100 | <0.001 | |
| Annual count | 1107 | 1229 | 1371 | 1475 | 1598 | 1639 | 1814 | 1898 | 1970 | <0.001 | |
| Ratio of weekly to annual count | 1.629 | 1.546 | 1.505 | 1.620 | 1.666 | 1.598 | 1.562 | 1.621 | 1.574 | 0.818 | |

**Supplementary Figure 1.**

**Temporal changes in the pathogenesis and classification of AF-related stroke.**
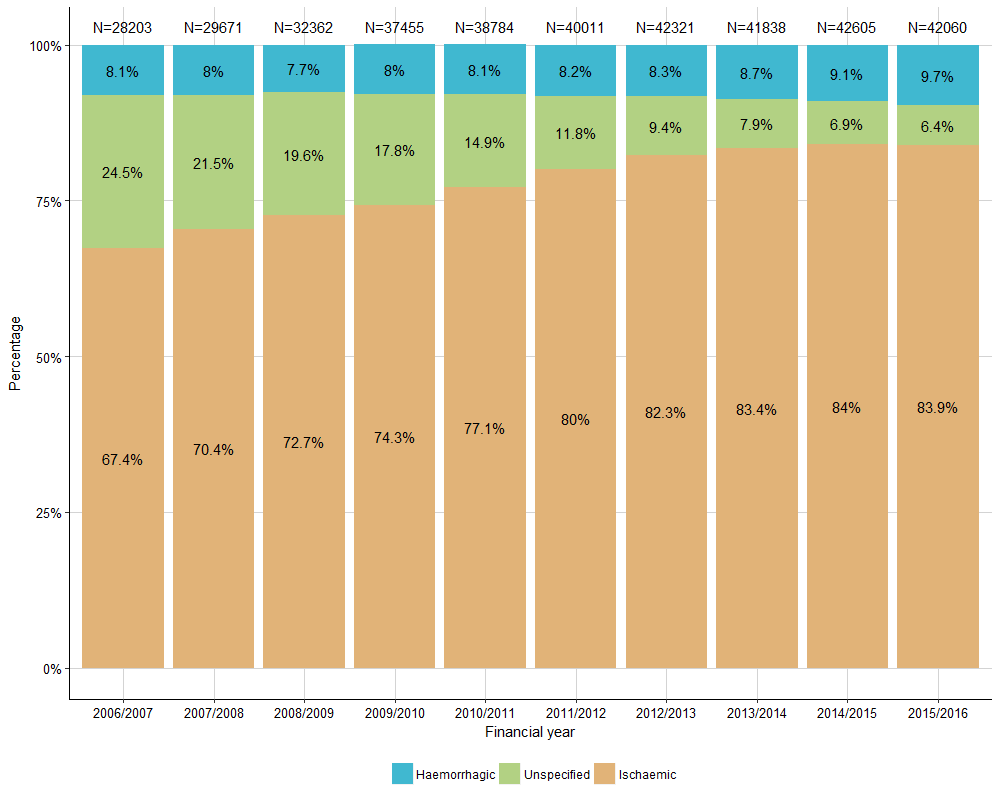


**Supplementary Figure 2. Comparison of weighted trends of anticoagulant and antiplatelet use based on CHADS_2_ and CHA_2_DS_2_-VASc scores.**

**
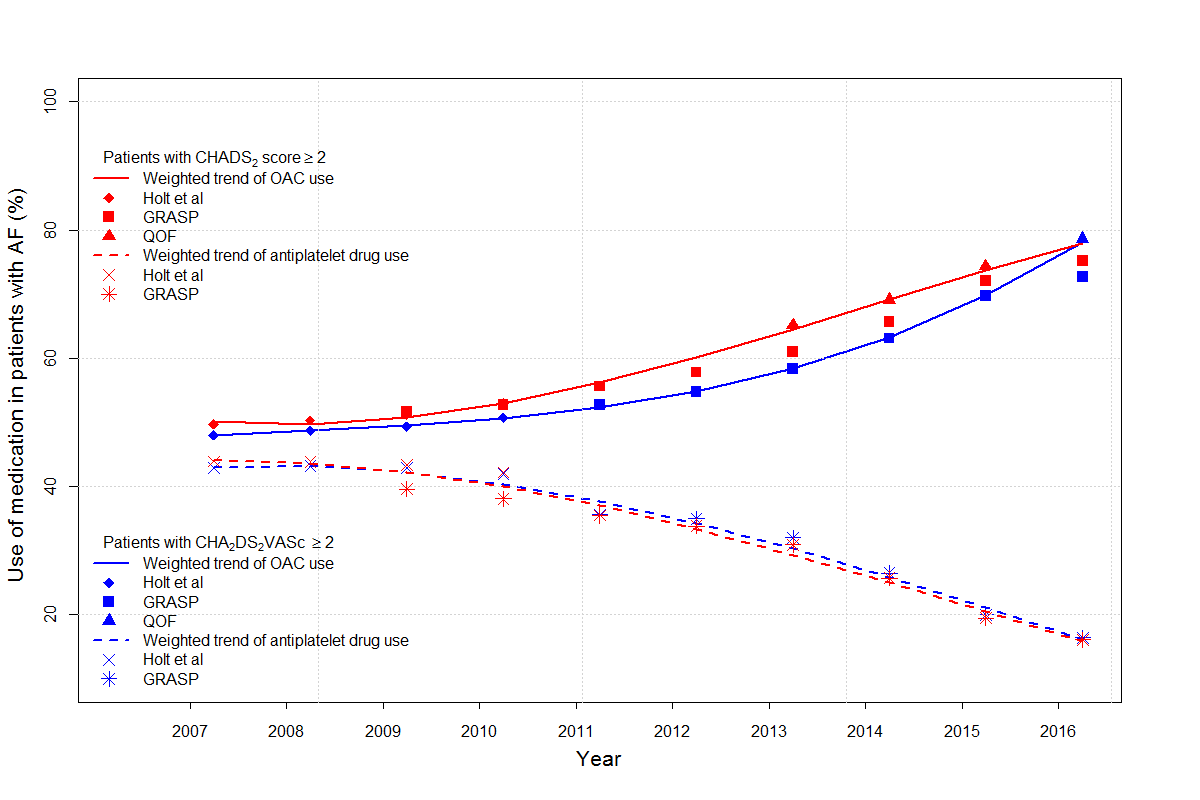
**

**Supplementary Figure 3.**

**The temporal trend of AF-related stroke stratified by stroke pathogenesis with and without a sensitivity analysis reallocating 90% of unclassified strokes as ischaemic and 10 % as haemorrhagic.**
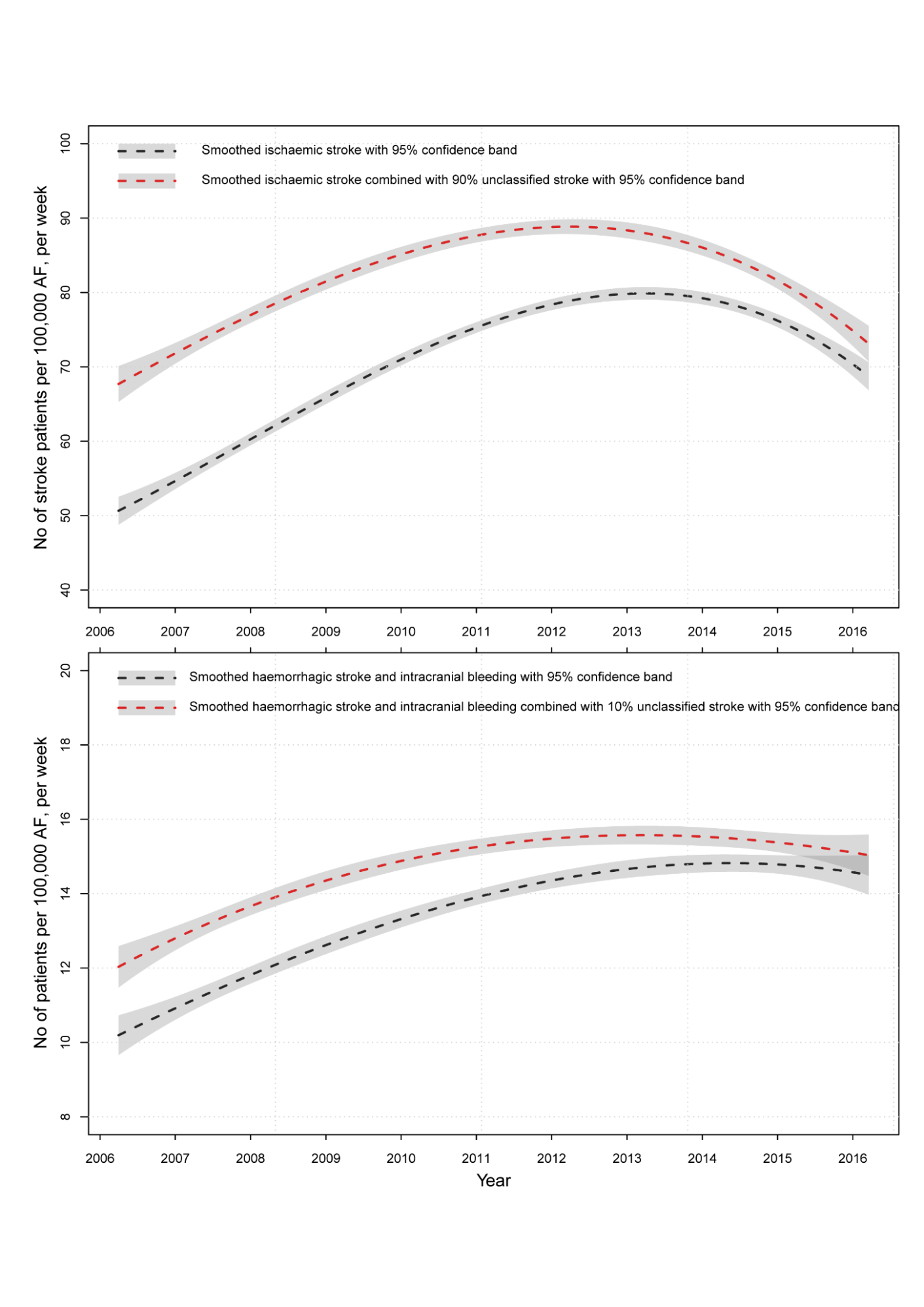


**Supplementary Figure 4.**

**Annual sum of weekly sampling timeframe counts and annual timeframe sampling counts of AF-related stroke**.


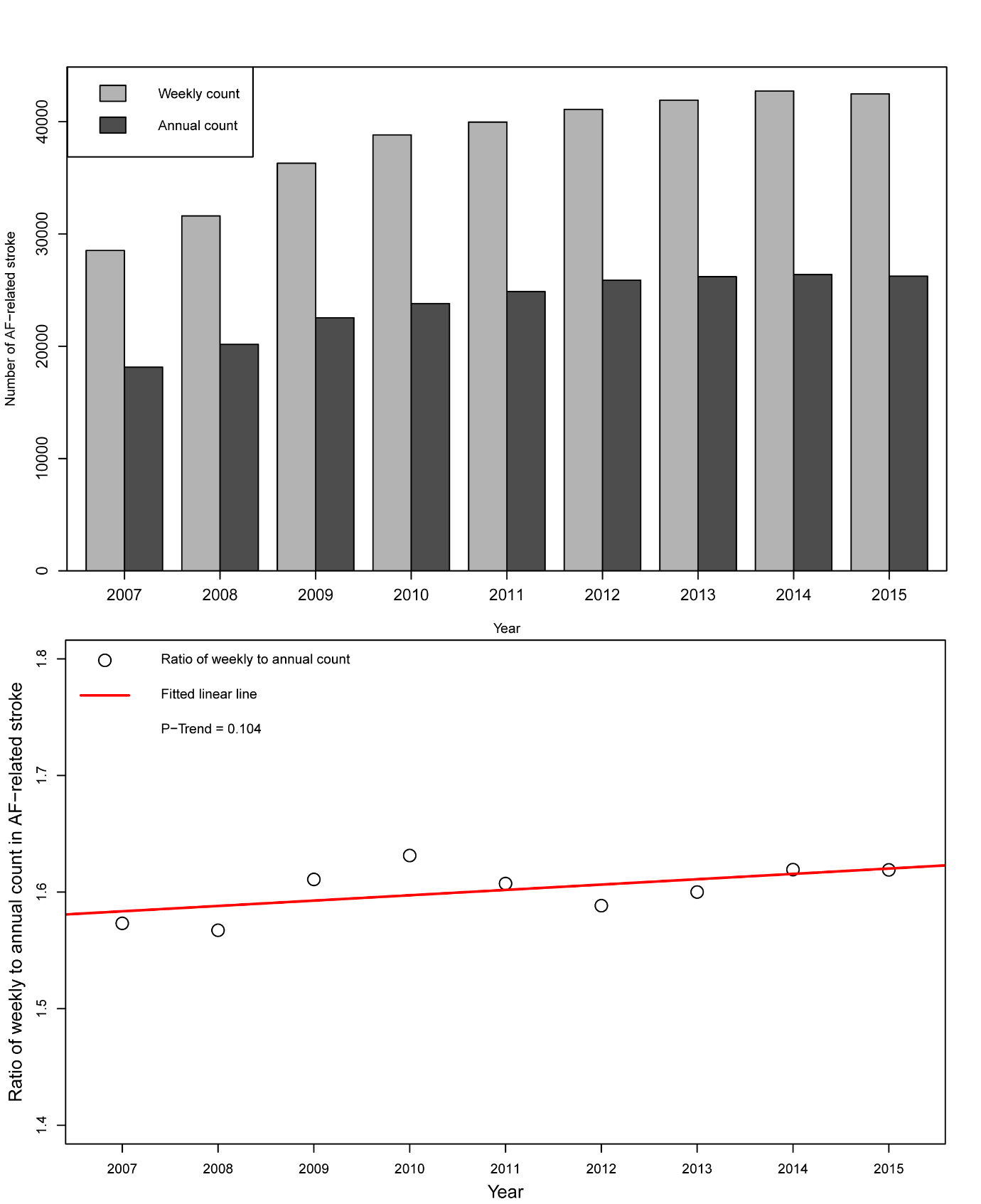


**Supplementary Section 2:**

**Magnitude of anticoagulant benefit**

An estimate was made of the number of strokes which would have occurred between April 2015 and April 2016 if the rate of anticoagulant uptake had stayed constant at the level of 1^st^ April 2009 (49%). Based on the observed association between anticoagulant uptake and stroke rate, the projected sum of weekly AF-related stroke counts between April 2015 and April 2016 would have been 48,805 (95% CI: 48,138–49,473 ). This compares with an observed count of 42,296 (95% CI: 41,663–42,929). The difference (6,335 episodes) between observed and predicted values includes replicates in HES between weeks. To make allowance for these replicates, this number was reduced by the weekly to annual ratio based on supplementary figure 3. On this basis, we estimate that an additional 4,068 AF-related incident strokes (95% CI 4,046–4,089) would have been expected in 2015/2016 if anticoagulant uptake had remained constant at 2009 levels.
